# Beyond Leptin and Adiponectin: The Diverse Roles of Adipokines in the Myocardial Hypertrophic Process and Heart Failure and Their Potential Contribution in Obesity

**DOI:** 10.3390/ijms27010003

**Published:** 2025-12-19

**Authors:** Morris Karmazyn, Xiaohong Tracey Gan

**Affiliations:** Department of Physiology and Pharmacology, University of Western Ontario, London, ON N6A 5C1, Canada

**Keywords:** adipokines, adipocytokines, obesity, cellular signalling, cardiac hypertrophy, cardiac remodelling, heart failure

## Abstract

It is now widely recognized that adipocytes have the ability to produce a myriad of bioactive compounds released into the circulation and affecting distal organs, including the heart. These factors, termed adipokines, are also produced by various tissues in addition to adipocytes, including cardiac tissue, and have the ability to modulate cardiac function and the response to pathology. Among the processes greatly affected by adipokines is myocardial remodelling due to hypertrophy and fibrosis, two processes that contribute to the development of heart failure. This is particularly relevant under conditions of obesity, and the accompanying increased adiposity, in general, results in increased adipokine production. The effects of adipokines on cardiac remodelling can be both beneficial or adverse, depending on the adipokine type, such as adiponectin and leptin, respectively. The molecular bases underlying the effects of adipokines on myocardial remodelling have been extensively studied and likely involve a multiplicity of cell signalling processes, thus demonstrating substantial complexity. Emerging evidence suggests that these proteins play an important role in cardiac pathology. Their precise contribution is yet to be determined with certainty, as this likely reflects a balance between pro-remodelling and anti-remodelling factors.

## 1. Introduction

It is now widely recognized that adipose tissue functions as an endocrine organ and possesses the capability to produce and release a plethora of bioactive proteins into the circulation that affect numerous aspects of human biology and homeostasis related to both physiology and pathophysiology. These proteins, termed adipokines, demonstrate an almost bewildering array of functions influencing numerous organ systems. More than 100 adipokines have thus far been identified, among which leptin and adiponectin arguably represent the most widely known of these factors. Adipokines, once released into the circulation, have the ability to modulate various disease processes by acting on specific receptors in distal organs in an endocrine manner, which results in alterations in cellular signalling. This is clearly the case with respect to the heart, which serves as a target for the actions of many adipokines and can indeed synthesize a variety of adipokines. These cardiac-derived adipokines can then function in an autocrine or paracrine manner to alter cardiac function. Nonetheless, adipocytes serve as the primary source of adipokines, and accordingly, the production of adipokines is greatly affected by the degree of adiposity, with generally increased levels seen in obese individuals.

Obesity is a major risk factor for the development of heart disease in general, including heart failure, which occurs as a result of myocardial remodelling due to cellular hypertrophy and increased fibrosis. The question arises as to whether adipokines participate in either a negative or positive manner in the development of heart failure, especially in obese individuals, where their production, at least with respect to most adipokines, is elevated. Is the increased risk of heart failure in obese subjects due to the production of adipokines? As discussed here, extensive evidence implicates adipokines in the myocardial remodelling process either by promoting myocardial remodelling or by functioning in a protective manner. It is widely believed that the balance between levels of pro- and antihypertrophic adipokines will determine their net effect on myocardial remodelling and the eventual development of heart failure.

A comprehensive understanding of the complexity of adipokines in terms of their mechanisms of action is rendered difficult by the sheer number of adipokines thus far identified and by the fact that each individual adipokine appears to affect multiple cell signalling and molecular events. Unravelling the key cellular mechanisms involved in the cardiac actions of adipokines represents a major challenge. In view of the emerging convincing evidence implicating adipokines in the heart failure process, here, we focus on a number of major adipokines and their potential roles in the development of heart failure, particularly, although not exclusively, as this relates to myocardial remodelling and heart failure associated with obesity and how this knowledge can be applied to therapeutic intervention.

## 2. Obesity and Heart Disease with Particular Emphasis on Myocardial Remodelling and Heart Failure

**Figure 1:**
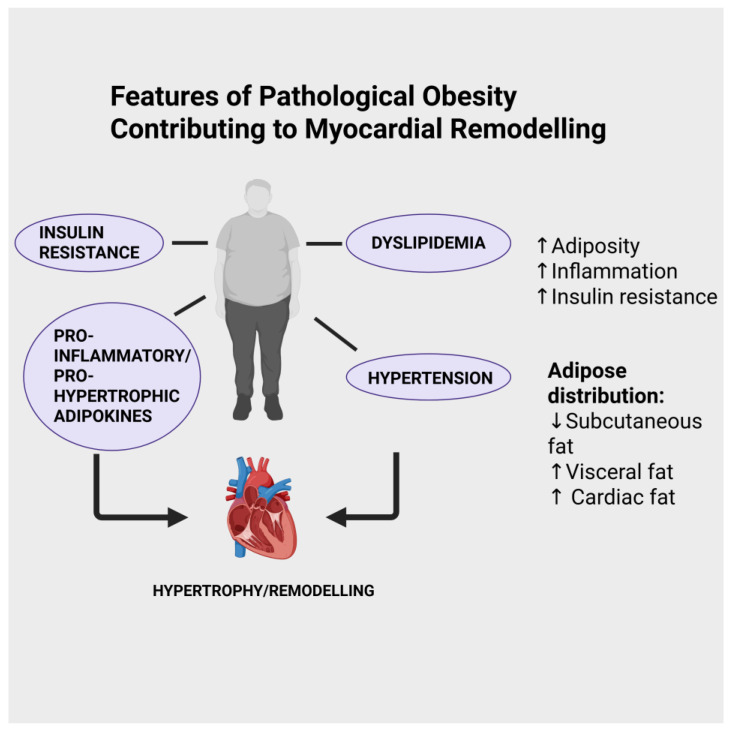
Some factors which contribute to myocardial remodelling and heart failure under conditions of obesity. Created in BioRender. Karmazyn, M. (2025) https://BioRender.com/ttojmt4.

The role of obesity as a major risk factor for many diseases has been recognized for many years (reviewed in [1,2]). Obesity, officially defined as those with a BMI of 30 kg/m^2^ or greater, is considered a disease by the WHO. According to World Health Organization (WHO) data, one in eight people in the world lived with obesity in 2022, and worldwide adult obesity rates have more than doubled since 1990 (https://www.who.int/news-room/fact-sheets/detail/obesity-and-overweight, accessed on 12 September 2025). One of the major consequences among various pathologies at increased risk in obese individuals is heart and other cardiovascular conditions, which encompass the development of heart failure [3,4]. While it is beyond the scope of this review to discuss the primary mechanisms that account for increased cardiovascular risk accompanying obesity in general, a number of well-established factors can be noted, as illustrated in Figure 1, including dyslipidemia, increased incidence of Type 2 diabetes, and hypertension [5]. Heart failure is a complex syndrome characterized by numerous features, including cardiac dysfunction, structural abnormalities, increased arrhythmogenesis, hypertension, cardiac lipotoxicity, insulin resistance, vascular endothelial injury, and others [6,7]. The release of biologically active factors from adipose tissue has been proposed as potentially regulating myocardial remodelling, although, as will be discussed in this review, this occurs in either a deleterious or beneficial manner. Many factors dictate the severity of cardiovascular risk under obese conditions, among these being the localization of the increased adiposity. For example, increased pericardial and visceral abdominal fat deposition has been identified as a particularly important risk factor for the development of cardiovascular disease, including heart failure [8,9,10,11,12,13]. Indeed, epicardial adipose tissue deposition has been identified specifically as a critical factor in the development of heart failure with preserved ejection fraction (HFpEF) [11,12]. Taken together, it is clear that the nature of body fat distribution is a critical factor when addressing the question of underlying mechanisms contributing to increased cardiovascular risk with obesity.

## 3. The “Obesity Paradox”: A Role for Adipokines?

Although obesity is generally considered an important contributor to heart disease including heart failure, the concept of the obesity paradox has been proposed by numerous authors and dictates that, in a subgroup of patients, obesity may be salutary and lead to a reduction in mortality in patients with heart failure [14,15,16]. The mechanisms underlying the obesity paradox are unclear but may be related to the nature and location of the adiposity, which could favour production of beneficial subcutaneous adipocyte-derived adipokines resulting in cardioprotection [17,18]. Indeed, as will be discussed here, a number of adipokines, including adiponectin and others, have been shown to have antiremodelling and antihypertrophic effects, and therefore, these could represent important contributors to the obesity paradox phenomenon.

While clearly representing an interesting and potentially important phenomenon helping to more fully understand the complex role of obesity in heart disease, it should be noted that others have questioned the concept of the obesity paradox and have shown that this proposed phenomenon is nonexistent when assessing obesity in subjects using newer anthropometric measurements of obesity rather than body mass index (BMI), such as waist-to-height ratio, relative fat mass, and body roundness index, and, additionally, when correcting for other prognostic variables [19]. Indeed, when applying these criteria to more than 1000 patients with heart failure with reduced ejection fraction, obesity was associated with increased mortality in these individuals [19]. Thus, the obesity paradox remains an overall conceptually interesting phenomenon that requires substantial future investigations to determine its validity and specifically, the potential contribution, if any, of “cardiac-friendly” adipokines.

## 4. Adipose Tissue as an Endocrine Organ

The concept of adipose tissue as a source of biologically active factors was first proposed in 1948 [20] and subsequently reviewed by a number of authors (e.g., [21,22,23]). Although leptin, first identified in 1994, holds an important distinction as the most studied adipose-derived factor to be identified with nearly 47,000 publications, likely due to its satiety influence, and one which exerts diverse and extensive biological effects, it is recognized today that adipocytes have the ability to produce and secrete a plethora of biologically active proteins generally referred to as adipokines or also adipocytokines. This knowledge has led to the genesis of a relatively novel and highly active area of biological research termed adipobiology, which has been proposed as a potential target for therapeutic intervention [24]. The science of adipobiology is based on the concept that adipose tissues, particularly white adipose tissues (WATs), are not simply structural units involved in fat storage but rather, as originally proposed by Wertheimer and Shapiro, also important modulators of energy metabolism [20]. Since the discovery of a myriad of adipokines, it is now widely accepted that adipocytes are, in effect, endocrine organs capable of modulating an extensive array of physiological functions through their release into the circulation of specific adipokines. It is interesting to point out that although leptin represents the most studied of the adipokines, it is not the first of such compounds to be identified—an honour that belongs to adipsin, discovered in 1987 (see below). As we shall note, these diverse effects of adipokines may represent both salutary and deleterious influences on biological properties, particularly with regard to the cardiac hypertrophic program. Although WATs are considered the primary source of adipokines, brown adipose tissues (BATs), considered primarily as regulators of thermogenesis, can also produce and release bioactive proteins into the circulation, such as neuregulin 4 (discussed below), known as batokines [25]. A concept important to underscore is that many adipokines, although considered primarily as adipocyte-derived actors, can also be produced by cells of non-adipocyte tissues. This is particularly the case with respect to the cardiovascular system, where some adipokines have been shown to be produced and released by the heart and vasculature, as noted below for individual adipokines.

However, as adipokines are principally derived from adipocytes, this has led to extensive research into their role, either beneficial or deleterious, in cardiac pathology, as well as pathologies of other organ systems in obesity, as the latter is obviously associated with increased adiposity and resultant dyslipidemia, which contributes to cardiovascular disease [26].

## 5. Obesity, Myocardial Remodelling and Heart Failure

An important issue to address is the nature of adipokine contribution to risk or severity of cardiac disease, including the development of heart failure seen in obese individuals, which occurs secondary to metabolic cardiomyopathy seen under conditions of obesity. As already noted, obesity itself has become a major world health concern. Data from the WHO show that one in eight people in the world was living with obesity in 2022 (Obesity and overweight fact sheet available from: https://www.who.int/news-room/fact-sheets/detail/obesity-and-overweight, accessed on 12 September 2025). Although long considered a major factor in heart disease development, the exact contribution of obesity is somewhat difficult to assign specifically in view of the underlying complex factors involved, as well as the potential role of the obesity paradox as a complicating factor in assessing the role of obesity in cardiovascular disease. In addition, the anatomical location of increased adiposity, age of the patient, and the existence of other risk factors can all ultimately contribute to the cardiovascular disease process in general [4] and, of relevance to this review, heart failure in particular. It appears that the primary heart failure subtype seen in obesity is diastolic dysfunction associated with heart failure with preserved ejection fraction [27]. In terms of fat distribution, excessive visceral fat is considered of particular importance in the development of heart failure [28], and clinical studies have shown that visceral adiposity, but not subcutaneous abdominal fat, even in the absence of heart failure was associated with concentric hypertrophic remodeling and significantly reduced left ventricular function [29]. In addition, epicardial adipose tissue may contribute to the development of heart failure via profibrotic and proinflammatory pathways [30,31].

The mechanisms by which obesity contributes to heart failure and cardiac dysfunction are varied and complex and involve defective energy metabolism and myocardial structure [32,33,34], disruption of autophagy [35], and many other metabolic and cellular defects [36,37]. Determining the role of adipokines and identifying the specific adipokines potentially involved in the development of heart failure is indeed a challenging task, particularly in view of the large number of adipokines thus far identified and the diverse and, indeed, opposing effects these adipokines exert on cardiovascular health [38]. As reasonably suggested by these authors, targeted interventions that affect distinct pathways would help to identify specific functions of individual adipokines affecting heart failure and potentially result in improved and novel therapeutic strategies [38].

## 6. Leptin and Adiponectin and the Critical Importance of the Leptin/Adiponectin Balance

**Figure 2:**
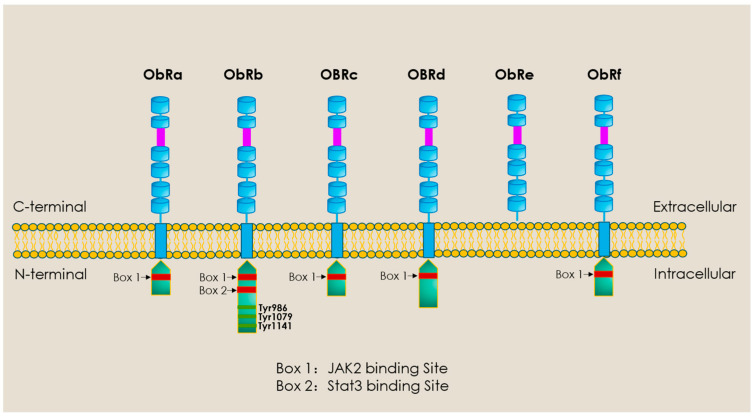
Diversity of leptin receptor subtypes. Of the six known isoforms, only ObRb expresses the full transmembrane region plus three tyrosine residues for full signalling activation, including activation of the JAK2/STAT3 pathways. Except for ObRe, shorter forms of ObR express only one intracellular domain (Box 1) and thus have limited signalling abilities. ObRe functions as a soluble leptin binding receptor not anchored to the cell membrane. Created with PowerPoint software (Microsoft Office 365).

When considering all adipokines thus far identified, it is evident that leptin, initially identified as an adipocyte-derived satiety factor by Jeffrey Friedman’s group in 1994 through cloning of the *ob/ob* gene [39], represents the most widely studied of these proteins not only with respect to appetite regulation and effects on energy balance but also as a modulator of the cardiovascular system with respect to its influence on disease processes in general and myocardial hypertrophy and heart failure specifically. The contribution of leptin to myocardial remodelling and the proposed underlying mechanisms have been reviewed in detail by the authors and other investigators. Accordingly, this will not be comprehensively discussed here, and interested readers are directed towards several recent reviews that discuss these issues [40,41,42,43]. Briefly, there is evidence that leptin, derived from either adipose, vascular, or cardiac tissue, contributes to the remodelling process in either a hormonal, paracrine, or autocrine manner [44,45]. Leptin exerts these effects by directly acting on specific receptors, which are expressed in the cardiomyocyte and other cardiac components such as fibroblasts. As illustrated in Figure 2, these receptors actually represent a family of six receptors termed ObR (or LepR), members of the Class 1 cytokine receptor family, which possess identical extracellular domains but differ markedly in the composition of their intracellular domain, dictating the nature of the intracellular signalling pathway. The only exception to this is the ObRe receptor, which functions as a soluble leptin binding receptor that is not anchored to the cell membrane and thus is unable to transduce any downstream cellular signalling. The ObRb receptor, which encodes the full intracellular domain, likely represents the primary receptor activating pro-remodelling cell signalling pathways. Although from a general perspective, ObRb activation leads to Janus Kinase 3-Signal Transducer and Activator of Transcription 3 (JAK2-STAT3) activation in many cells [46], stimulation of non-JAK2-dependent pathways has also been documented [47]. Indeed, as summarized in Table 1, the pro-remodelling/hypertrophic effect of leptin has been linked to activation of various intracellular signalling mechanisms.

Adiponectin, initially referred to as Acrp30 (for adipocyte complement-related protein of 30 kDa), was first identified in 1995 [60]. It is also the most abundant of the known adipokines in terms of serum concentrations, and interestingly, its plasma concentration inversely correlates with the degree of adiposity [61,62]. Adiponectin produces its effects by binding to its receptors, termed AdipoR1 or AdipoR2 [63], with both receptors, and it is shown to be expressed in adult ventricular myocytes and linked to AMP-activated protein kinase (AMPK) activation [64]. Indeed, AMPK activation and the resultant subsequent suppression of extracellular signal-regulated kinase (ERK) activation have been proposed as primary mechanisms underlying the antihypertrophic effect of adiponectin involving both AdipoR1 and AdipoR2 activation [65,66]. However, of the two receptors, AdipoR1 likely represents the primary receptor subtype underlying the antihypertrophic effect of adiponectin. For example, patients expressing an AdipoR1 mutation demonstrate hypertrophic cardiomyopathy, whereas the overexpression of mutant AdipoR1 in cultured cardiomyocytes or the expression of the mutant receptor in transgenic mice leads to hypertrophic responses in the absence of any other intervention [67]. These hypertrophic responses were shown to be mediated via p38/mTOR and/or ERK1/2 pathways and reversed by rapamycin [67]. Moreover, adiponectin knockout mice demonstrate an enhanced hypertrophic response to pressure overload produced by thoracic aorta banding [65]. Accordingly, adiponectin and leptin could be considered as adipokines exerting opposite effects on the cardiovascular system. As summarized in Table 2, evidence for a direct antihypertrophic effect of adiponectin is quite extensive based on studies using a variety of experimental models.

The studies listed in Table 2 support the concept of a salutary antihypertrophic effect of adiponectin under various experimental conditions. Interestingly, the hypertrophic response in mice subjected to pressure overload is enhanced in adiponectin null mice through a mechanism associated with the enhanced activation of the prohypertrophic transcriptional factor myocyte enhancer factor-2 (Mef2), thus suggesting that background adiponectin is required for the induction of cardiac hypertrophy following induction of pressure overload [78]. Moreover, adiponectin deletions have been shown to exacerbate heart failure in mice subjected to thoracic aorta constriction, thus further implicating adiponectin as a protective factor [79].

## 7. Omentin

Omentin was first identified nearly 20 years ago as a novel glycoprotein adipokine produced in omental visceral (hence its name) adipocytes, playing a potentially important role in regulating the actions of insulin and thus glucose homeostasis [80]. This adipokine has two isoforms, namely, omentin-1 and omentin-2, with the former demonstrating the greater abundance and biological effects [80,81]. Omentin-1 is generally considered a beneficial agent in view of its ability to protect vascular endothelium, dilate blood vessels, and improve glucose homeostasis, as well as possessing anti-inflammatory properties [82]. There is evidence that some of the beneficial actions of omentin-1, at least with respect to its direct hypotensive effects in normotensive rats, may be dependent on increased production of adiponectin with a resultant increased nitric oxide production [83].

With respect to myocardial remodelling and heart failure, there is emerging evidence that omentin functions as an antihypertrophic factor following myocardial insult and that it may serve as a significant predictor of the development of left ventricular hypertrophy, at least in patients with Type 2 diabetes [84]. Among the first studies to demonstrate this effect was presented by Matsuo et al. [85], who showed that administration of an adenoviral vector expressing human omentin to mice subjected to transverse aortic constriction or angiotensin II administration resulted in attenuation of hypertrophy and fibrosis, as well as ERK phosphorylation. A similar effect was seen in cultured cardiomyocytes treated with phenylephrine, in which human omentin protein effectively reduced the hypertrophic response [85]. The authors attributed the antihypertrophic effect of omentin to increased phosphorylation of AMPK. Indeed, the prevention of AMPK phosphorylation prevented the antihypertrophic effect of omentin [85].

Omentin was also shown to reduce hypertrophy in another in vivo model comprising sustained coronary artery ligation. In this regard, transgenic mice expressing the human omentin gene in adipocytes demonstrated a higher survival rate and reduced hypertrophy and fibrosis 4 weeks after induction of infarction compared to their wild-type cohorts [86]. Moreover, omentin-expressing transgenic mice demonstrated improved left ventricular function compared to wild-type mice, as determined by echocardiography [86]. These results were also apparent in wild-type mice treated 3 days post-coronary ligation with intravenous administration of adenoviral vectors expressing human omentin [86]. Interestingly, omentin-1 was also found to inhibit the prohypertrophic effect of the adipokine resistin (discussed in the following section) in cultured H9c2 cardiomyoblasts [87]. This effect was attributed to inhibition of resistin-induced upregulation of pro-inflammatory signalling involving the (Toll-like receptor 4/Myeloid differentiation factor 88/Nuclear factor kappa-light-chain-enhancer of activated B cells (TLR4/MyD88/NF-κB) phosphorylation pathway [87].

The clinical relevance of omentin to heart failure or myocardial remodelling remains to be determined with certainty, although omentin-1 levels were shown to be decreased in elderly patients with HFpEF [88]. Similar results were reported by Huang et al. [89], who demonstrated reduced plasma omentin-1 levels in patients with heart failure associated with dilated cardiomyopathy. Thus, based on these findings, it has been suggested that plasma omentin-1 levels may represent a suitable marker for the development of heart failure in the clinical scenario. However, omentin-1 levels appear to be only mildly associated with left ventricular dysfunction in heart failure patients based on linear regression analyses [90].

## 8. Resistin

The cysteine-rich adipokine resistin was first identified in 2001 and is so named due to its ability to increase insulin resistance when injected into experimental animals [91]. Of potential relevance to cardiac pathology, resistin, albeit along with other proinflammatory adipokines, has been shown to increase expression of proinflammatory factors [92,93]. Resistin is expressed in the heart [94], and there is abundant evidence that resistin contributes to the myocardial remodelling process even in the absence of any pathological insult. Thus, myocardial overexpression of resistin produces a multifaceted phenotype consisting of oxidative stress, inflammation, fibrosis, apoptosis, hypertrophy, and cardiac dysfunction, 9 weeks following adenoviral transfection in rats [95]. Adipose tissue-specific deletion of resistin reduced myocardial remodelling and improved cardiac function in mice subjected to pressure overload-induced aortic banding produced by 10-week thoracic aorta coarctation, while, in contrast, resistin overexpression exacerbated the deleterious effect of pressure overload [96]. These effects were proposed to occur via modulation of the expression of micro RNAs (miRNAs), which are important for post-transcriptional regulation of key factors involved in the hypertrophic and remodelling processes. In this particular case, pressure overload was associated with increased expression of miR148b-3p leading to DNA damage, which was attenuated by resistin inhibition and increased with overexpression of the adipokine [96]. Resistin has also been implicated as a major contributor to diabetic cardiomyopathy [97], although this may reflect its ability to mediate enhanced hyperglycemia through increased insulin resistance under diabetic conditions [91].

While the above studies show that resistin can augment the remodelling response to pathological insult, there is evidence that cardiomyocyte overexpression per se can directly alter cell characteristics, although this appears to vary in terms of molecular and cellular mechanisms underlying this response. Thus, adenovirus-mediated overexpression of resistin in cultured neonatal rat ventricular myocytes produced a hypertrophic response and activation of signal transduction pathways, including MAPK signalling [94]. Overexpression of resistin in adult cultured rat cardiomyocytes significantly depressed cell shortening associated with defective intracellular calcium homeostasis [94]. Direct addition of resistin has been shown to produce hypertrophy in cultured H9c2 cardiac myoblasts, which was attributed to inactivation of the liver kinaseB1 (LKB1)/AMPK cell signaling pathway [98]. Moreover, direct addition of resistin to H9c2 myoblasts produced hypertrophy, which was inhibited by omentin through inhibition of the TLR4/MyD88/NF-κB signalling pathway [87]. Similar opposing effects of adipokines were shown with apelin (discussed in Section 10), which reduced the prohypertrophic effect of resistin in H9c2 myoblasts via ERK1/2 inhibition [99]. These studies reinforce the concept that the net effect of adipokines on myocardial hypertrophy and remodelling will reflect the balance of pro- and anti-hypertrophic adipokines produced. Overexpression of resistin in NRCMs, as well as in rats, produced a hypertrophic response associated with AMPK inhibition and activation of mTOR and its downstream target ribosomal protein S6 kinase beta (p70S6K), as well as the apoptosis signal-regulating kinase 1/c-Jun N-terminal Kinase pathway [100]. Moreover, overexpression of cardiac human resistin produced right ventricular dilatation and dysfunction, suggesting that resistin can contribute to right ventricular dysfunction in response to pulmonary hypertension [101]. Cardiac dysfunction associated with resistin overexpression was attributed to suppression of protein kinase A and AMP-activated protein kinase [101]. Right ventricular tissue of pulmonary hypertension patients demonstrates increased resistin expression, as does right ventricular tissue from rats in which pulmonary hypertension was induced by monocrotaline treatment [101].

From a clinical perspective, serum resistin levels have been positively correlated with poor prognosis in patients with heart failure, severity of heart failure [102], and risk for developing heart failure in elderly subjects, possibly involving the induction of proinflammatory cytokines [103,104]. This association between resistin and increased heart failure risk was also seen in younger subjects (*N* = 2739) as part of the Framingham Offspring Study, with no relationship observed with respect to adiponectin and heart failure [105], the latter finding in contrast to other reports discussed earlier in this review. It is possible that this lack of association between adiponectin and heart failure reflects the occurrence of adiponectin resistance seen in patients with advanced heart failure [106]. Although a more recent study showed a clear association between serum resistin levels and incident heart failure, no association was observed in relation to subclinical myocardial fibrosis [107].

## 9. Visfatin

Visfatin is an interesting adipokine that has undergone a number of name modifications. It was first identified in 1994 as a pre-B-cell colony-enhancing factor [108]. Subsequently, it was found that the pre-B-cell colony-enhancing factor had a critical role in NAD biosynthesis, thereby regulating the deacetylase activity of sirtuins, and was thus renamed nicotinamide phosphoribosyltransferase (NAMPT) [109]. NAMPT was found to be secreted by visceral adipocytes and renamed visfatin for “visceral fat-specific adipokine” [110,111]. To enhance clarity and maintain consistency, visfatin will be used to denote this adipokine in the present discussion. Although visceral fat tissue represents the primary source of visfatin expression and production, the protein is generally ubiquitously expressed and has been identified in cardiomyocytes, and its expression can be induced by a number of factors, including angiotensin II and glucose [112,113]. The expression of visfatin has been shown to be under the control of the nuclear receptor REV-ERB complex [114]. Moreover, visfatin is expressed in cardiac fibroblasts, which may be of relevance in terms of its role in myocardial remodelling [115], as discussed further below. In clinical studies, plasma visfatin levels have been shown to be associated with an increased incidence of major adverse cardiovascular events in myocardial infarction patients [116].

Studies vis-à-vis cardiac effects of visfatin suggest that it is a beneficial adipokine, as initially shown in terms of its ability to produce a hypoglycemic effect under obesity or type 2 diabetes [117]. Moreover, studies have shown that visfatin administration to mice subjected to myocardial infarction followed by reperfusion reduces infarct size in these animals [118]. These effects may be due to visfatin-induced increased NAD and ATP production in the cardiomyocyte, leading to increased cell survival via a reduction in apoptosis and stimulation of autophagy [119]. Further evidence for a beneficial role of visfatin comes from studies demonstrating an increased extracellular monocyte-derived circulating visfatin level in pressure-overloaded mice subjected to thoracic aortic banding [120]. Importantly, pharmacological inhibition of visfatin by FK866 increased cardiomyocyte apoptosis and cardiac decompensation in these animals [120].

While stimulation of autophagy is considered a potential mechanism for reduced postinfarction remodelling, visfatin has paradoxically been shown to directly stimulate collagen synthesis and proliferation of cultured cardiac fibroblasts through a mechanism involving p38MAPK, phosphatidylinositol 3-kinase (PI3K), and ERK1/2 pathways, thus potentially contributing to enhanced myocardial remodelling [121]. Indeed, transgenic mice overexpressing visfatin demonstrated pronounced spontaneous cardiac hypertrophy 6 months after treatment in the absence of other prohypertrophic interventions [122]. The hypertrophic response was associated with various intracellular signalling pathways, including activation of mitogen-activated protein kinases, c-Jun N-terminal kinase (JNK)1, p38, and ERK, as well as increased calcineurin levels and nuclear factor of activated T cells (NFAT) translocation to nuclei [122]. A prohypertrophic effect of visfatin has also been demonstrated in a study using H9c2 fibroblasts, where exposure of these cells for up to 72 h resulted in a hypertrophic response proposed to be mediated via an endoplasmic reticulum stress and transient receptor potential canonical channel 1 (TRPC1) pathway [123]. Visfatin administration has also been found to increase cardiomyocyte hypertrophy, increase cardiac fibrosis, and reduce cardiac function when administered to mice subjected to thoracic aorta banding [124]. These deleterious effects of visfatin were found to be associated with a macrophage-dependent increase in oxidative stress [124]. Evidence for a deleterious role of visfatin was presented in a recent study in which a visfatin-neutralizing antibody reduced remodelling and improved cardiac function in rats subjected to a sustained 4-week myocardial infarction via inhibition of the visfatin/TLR4 inflammatory cascade [125].

It has also been proposed that visfatin may play a beneficial role in mediating reverse myocardial remodelling seen following the debanding of aortic-banded mice or in patients with aortic stenosis following aortic valve replacement [126]. This concept was suggested based on myocardial visfatin measurements, which were significantly reduced both during aortic banding in mice or stenosis in patients, whereas values increased following debanding or aortic valve replacement [126]. Although this study provides solely indirect evidence, the authors suggest that visfatin may be an important regulator of myocardial remodelling due to pressure overload. Moreover, visfatin upregulation has been shown to protect the heart against the development of cardiomyopathy in a high-fat diet-induced mouse diabetes model consisting of a mechanism involving an NAD^+^-dependent anti-oxidative stress process [127].

As noted above, visfatin is also expressed in cardiac fibroblasts and upregulated, along with Type 1 procollagen, under high glucose (30 mmol/L compared to 5.5 mm/L for control conditions) exposure through a mechanism involving the ROCK signalling pathway [115]. Although exposure of fibroblasts to high glucose conditions enhanced fibroblast proliferation, a potential role of visfatin was not studied [115].

When taken together, it is apparent that visfatin plays complex and multifaceted roles in the development of heart failure, possibly dependent on the experimental model. This has been strongly suggested in a study using mice subjected to thoracic aortic banding, i.e., pressure overload, where it was shown that endogenous visfatin protects the heart in this model, although its cardiac-specific overexpression enhances myocardial hypertrophy, fibrosis, apoptosis, and mitochondrial energy dysfunction and reduces cardiac function as assessed by echocardiography [128]. Loss of cardiac visfatin per se in the absence of any other insult produces a heart failure phenotype consisting of metabolic derangements, myocardial remodelling, and sudden cardiac death, which were dependent on NAD^+^, thereby suggesting a critical role for endogenous visfatin for the maintenance of normal cardiac function [129].

## 10. Apelin

The adipokine peptide apelin was first identified in the bovine stomach [130] and subsequently shown to exert its effects through the activation of the G-protein-coupled APJ receptor. First identified in 1993, this receptor is interesting in that it shares approximately a 50% homology with the angiotensin II type 1 (AT1) receptor [131] although it is not activated by angiotensin II. Indeed, APJ activation negatively regulates AT1 receptor activation through negative allosteric regulation [132], and apelin has been shown to exert effects opposite to those seen with angiotensin II and protect against angiotensin II-induced cardiac fibrosis [133]. Although apelin is generally considered as an antihypertrophic factor, as discussed below, there is evidence that the APJ receptor per se may function as a dual receptor, both promoting and inhibiting the hypertrophic response. In this regard, Scimia and coworkers [134] have shown that APJ knockout mice demonstrate a reduced hypertrophic response in response to sustained thoracic aorta binding, whereas mice lacking apelin retain the hypertrophic response, thus suggesting an apelin-independent role of APJ. The authors’ primary conclusion invokes a stretch-induced activation of the APJ, resulting in a hypertrophic response via a β-arrestin dependent mechanism, whereas apelin confers an APJ-dependent antihypertrophic effect by activation of G-protein Gαi [134]. Overall, APJ appears to play diverse roles in cardiac intracellular, and the receptor has been shown to function as a sensor in response to myocardial stretch, resulting in a positive inotropic response [135].

Apelin is initially secreted as a 77-amino acid preproprotein, which is subsequently cleaved to shorter fragments. The most abundant and important of these fragments in terms of biologic effects is the 13-amino acid apelin-13 fragment, which has been identified both in plasma [136] and the human heart [137].

Recently, a second endogenous ligand for the APJ receptor named Elabela, a bioactive micropeptide, has been identified and appears to play important roles in numerous physiological processes [138]. Elabela has been shown to exert numerous cardiobeneficial effects, including reduction in oxidative stress-induced injury [139] and a reduction in pressure-overload-induced cardiac dysfunction and hypertrophy produced by angiotensin II infusion in mice [140]. Elabela has also been reported to improve cardiac function and reduce hypertrophy in heart failure produced by myocardial infarction in mice through a mechanism involving vascular endothelial growth factor receptor 3 activation [141]. While the beneficial results of Elabela are somewhat predictable based on the ability of apelin to reduce hypertrophy and heart failure via APJ activation, it is interesting that some differences between Elabela and apelin have been reported. One such area involves the enzyme angiotensin converting enzyme 2 (ACE2), a homolog of ACE1 and a negative regulator of the renin angiotensin system (RAS) by cleaving angiotensin II, and thus, it is generally considered as an inhibitor of myocardial remodelling [142]. Apelin has been shown to increase ACE2 levels in the failing heart and to couple apelin to RAS [143], and it is interesting to add that the activation of the Apelin-ACE 2 pathway has been proposed as a mechanism for the beneficial effects of the sodium–glucose cotransporter-2 (SGLT2) inhibitor canagliflozin in a model of hypertrophy and heart failure in salt-sensitive rats [144]. This effect of apelin also reveals differences between apelin and Elabela. For example, when comparing the effects of apelin and Elabela in the study just cited [140], it was shown that apelin treatment did not modulate ACE mRNA expression but upregulated ACE2 expression in pressure-overloaded hearts. In contrast, Elabela did not affect ACE2 expression but downregulated ACE expression. Thus, while both AJP agonists reduced the effect of pressure overload equally, this was likely mediated by different effects on the angiotensin system [140].

Studies into the cardiac influence of apelin have primarily, but not exclusively (see below), demonstrated beneficial effects, including reduction, in ischemic and reperfusion injury [145], reduction in blood pressure through nitric oxide generation [146], improved myocardial repair following myocardial infarction [147,148], increased myocardial vascular density in a mouse model of diabetic cardiomyopathy [149], and attenuation of atrial fibrosis, which can contribute to the development of atrial fibrillation [150]. Thus, targeting the apelin system is considered a potentially effective approach towards mitigating a number of cardiovascular disorders, including cardiac hypertrophy [151,152]. In terms of heart failure, cardiac apelin expression has been shown to be reduced in experimental left ventricular hypertrophy and heart failure comprising Dahl salt-sensitive rats, which was reversed by treatment with the AT1receptor blocker telmisartan [153]. Moreover, apelin knockout has been shown to increase the prohypertrophic and proremodelling effects of angiotensin II infusion and enhance impaired contractility in aging mice [154]. Although most studies point to depressed apelin levels in the hypertrophied myocardium, plasma levels are increased, as demonstrated in both animal and human studies, which the authors attributed to a compensatory mechanism aimed at blunting the hypertrophic response [155]. However, hypertensive patients with left ventricular hypertrophy were shown to exhibit reduced plasma apelin levels when compared to hypertensive subjects without left ventricular hypertrophy [156]. The differences between myocardial vs. plasma apelin levels have been attributed to the fact that failing hearts release apelin into the circulation in contrast to apelin uptake by non-failing hearts [157].

Apelin can also produce beneficial effects through its positive inotropic influence [158,159] and afterload reduction [159], and clinical studies have shown that apelin-13 infusion improves cardiac function in heart failure patients [160,161]. Apelin has also been shown to reduce the severity of acute heart failure in rabbits as a result of sodium pentobarbital administration through a mechanism involving reductions in endoplasmic reticulum stress [162], whereas a selective APJ receptor agonist improved indices of cardiac function in the renal hypertensive rat model of cardiac hypertrophy and reduced cardiac output [163]. In addition to the beneficial effects of apelin-13, a metabolically resistant analogue of apelin-17 has been shown to reduce myocardial remodelling and improve ventricular function in a murine model of heart failure produced by sustained coronary artery ligation [164]. Of particular relevance to obesity, apelin has been shown to reverse the direct hypertrophic effect of a 24-week high-fat diet in mice when administered for two weeks following high-fat diet cessation [165]. This effect was associated with various improved indices, including cardiac function and improved intracellular Ca^2+^ homeostasis, endoplasmic reticulum stress, and autophagy [165]. Moreover, apelin prevents the development of heart failure in mice fed a high-fat diet and subjected to pressure overload via aortic banding [166]. These effects of apelin were associated with improved myocardial energy metabolism, as evidenced by improved fatty acid utilization and glucose oxidation, as well as reduced mitochondrial injury [166].

Apelin can also reduce myocardial remodelling by a reduction in fibroblast proliferation. Indeed, treatment of mouse cardiac fibroblasts with apelin reduced fibroblast activation and collagen production, which was mediated by a reduction in sphingosine kinase 1; this was similarly observed in an in vivo model of heart failure produced by aortic banding [167].

Other mechanisms by which apelin could reduce the hypertrophic response to various factors are likely multifactorial, but the antihypertrophic effect has been shown to be related to reduced oxidative stress by increased myocardial catalase activity [168], as well as a potential reduction in the inflammatory response, as demonstrated by a reduction in inflammation-associated markers in cultured cardiomyoblasts [169].

Based on numerous positive results using apelin in heart failure models, the peptide shows promise as a potential therapeutic agent, although its effectiveness is limited by a short in vivo half-life of less than 5 min [170]. This limitation can be overcome by developing stable analogues of apelin that are not readily degradable or by identifying novel delivery strategies. For example, with respect to the latter, it has previously been shown that apelin encapsulation in liposome nanocarriers conjugated with polyethylene glycol (PEG) polymers produced sustained apelin release and improved cardiac function in a mouse model of pressure-overload induced heart failure produced by14-day thoracic aortic banding [171].

As briefly noted above, although cardiac antihypertrophic effects of apelin have been extensively demonstrated, contrary findings have also been reported. Thus, the same group has consistently demonstrated that apelin can induce hypertrophy in cultured H9c2 rat myoblasts by stimulating autophagy pathways through mechanisms associated with increased PI3K phosphorylation [172,173], as well as caveolin-1 inhibition [174], and increased endoplasmic reticulum stress [175]. These findings add substantial complexity to our understanding of apelin and cardiac hypertrophy, although it should be appreciated that myoblasts may respond differently to various stimuli when compared to effects on ventricular myocytes.

## 11. Vaspin

The 47 kDa 415 amino acid adipokine vaspin (so named from visceral adipose tissue-derived serpin) was first identified in 2005 in visceral adipose tissue of obese and Type 2 diabetic rats and proposed to function beneficially to reduce insulin resistance [176]. Our current knowledge of the relevance of vaspin to cardiac health is somewhat limited, although low plasma concentrations have been shown to be associated with increased major cardiac events, including heart failure, in patients following acute myocardial infarction [177].

A small number of studies have investigated the effect of vaspin on cardiac hypertrophy experimental models, with uniform findings showing beneficial effects in terms of reducing hypertrophy and myocardial remodelling. For example, vaspin was shown to suppress myocardial fibrosis and improve cardiac function in mice subjected to heart failure produced by either myocardial infarction, thoracic aortic banding, or angiotensin II infusion, each treatment for a period of four weeks [178]. These beneficial effects were attributed to a reduction in oxidative stress subsequent to suppression of the PI3K/Akt (protein kinase B) pathway [178]. A more recent study showed that vaspin-knockout mice demonstrate a greatly enhanced myocardial remodelling and hypertrophy, as well as ventricular dysfunction produced by 9-day isoproterenol administration, thereby further demonstrating an antihypertrophic effect of vaspin [179]. Based on these studies, as well as studies using cultured neonatal cardiomyocytes, the authors demonstrated a key role for enhanced PI3K/Akt/mTOR pathway-dependent autophagy as principally accounting for vaspin-induced protection [179]. Interestingly, vaspin has also been shown to reduce cardiomyopathy and myocardial remodelling in a mouse model of lipoatrophy via stimulation of Akt phosphorylation [180].

## 12. Adipsin

The discovery of the adipokine adipsin, also known as complement factor D, in 1987 predates the identification of leptin as a protein produced by adipose tissue [181]. Adipsin plays an important role in the complement system primarily by serving as the rate-limiting factor in the alternative complement pathway [182]. In addition, adipsin is important in the regulation of pancreatic insulin release by promoting insulin release [183] via the C3a/C3a receptor 1 [Complement C3a receptor 1 or C3aR1] pathways, as well as protecting pancreatic beta cells under diabetic conditions (reviewed in [184]).

Although adipsin has been implicated as a potential contributor to various cardiovascular disease, including those associated with vascular dysfunction (reviewed in [185]), at present, there is a paucity of data related to its potential role in myocardial remodelling or the development of heart failure. Substantial studies need to be carried out in order to elucidate the potential role of this adipokine in this form of pathology.

## 13. Asprosin

Asprosin is a relatively novel adipokine first discovered in 2016 as a glucogenic hormone that is recruited primarily to the liver, where it produces rapid glucose release through the activation of the G-protein-dependent cyclic adenosine monophosphate (cAMP)/PKA pathway [186]. The injection of asprosin to mice results in a rapid increase in blood glucose, suggesting that targeting the asprosin pathway could be therapeutically effective for the treatment of diabetes mellitus [186].

Asprosin expression has been identified in rat cardiomyocytes and found to be decreased along with serum levels of the adipokine in animals subjected to STZ-induced diabetes [187]. In terms of cardiac remodelling and heart failure, one clinical study has suggested that enhanced asprosin plasma levels may be associated with a reduction in adverse events in patients with dilated cardiomyopathy [188]. Based on studies using hypoxic cultured cardiac myoblasts treated with asprosin, the authors attributed the beneficial effects to increased cell viability concomitant with improved mitochondrial function [188]. The use of SGLT-2 inhibitors such as empagliflozin for the treatment of heart failure patients has been shown to be associated with elevated serum asprosin levels, suggesting that asprosin may account, at least in part, for the beneficial effects of these drugs [189].

While the findings noted above suggest a beneficial role of asprosin, a recent report demonstrated that circulating asprosin concentrations are increased in heart failure patients and that elevated levels of this adipokine were positively correlated with the incidence of heart failure, leading the authors to suggest that elevated asprosin may be a risk factor related to the development of heart failure [190]. This finding is supported by the observation that elderly patients with Type 2 diabetes and left ventricular diastolic dysfunction exhibited elevated asprosin serum levels when compared to patients without left ventricular dysfunction [191]. Moreover, increased serum levels of asprosin were associated with increased risk of developing left ventricular dysfunction in these patients [191].

## 14. Chemerin

Chemerin, which is also known as tazarotene-induced gene 2, was originally identified in 1997 in psoriatic skin lesions [192] and proposed to function as an immunomodulator and to play an important role in a number of pathologies [193,194]. Although chemerin expression has been identified in various tissues, including visceral adipose tissue, placenta, and liver, it has also been identified in the heart [195]. In terms of heart disease, serum chemerin levels are elevated in patients with coronary artery disease, suggesting that this adipokine could serve as a useful marker and predictor for coronary disease and possibly other cardiovascular events [196]. Moreover, serum chemerin levels have been shown to be a predictor of adverse cardiac events in patients with heart failure [197]. Its role in the development of heart failure and myocardial remodelling is at present unknown, although at least one of the three receptors involved in chemerin cell signalling, C-C chemokine receptor-like 2 (CCRL2), has been identified in the heart and shown to participate in experimental autoimmune myocarditis in mice [198]. Although substantial future research is necessary in order to fully delineate the role of chemerin in cardiac pathology, including myocardial hypertrophy and heart failure, there is the potential of targeting the chemerin system as a therapeutic strategy for a number of cardiovascular pathologies [199].

## 15. Meteorin-like Protein

Meteorin-like protein (Metrnl) is so named as it shares substantial amino acid sequence homology with the central nervous system-specific neurotrophic factor Meteorin [200]. Metrnl has been shown to play important roles in immunology, inflammation, and metabolism, such as improved glucose metabolism [200]. As such, it likely contributes to a variety of pathologies and may serve as a target for therapeutic interventions or as a treatment modality for various conditions such as muscle regeneration [200,201]. Unlike Meteorin, Metrnl is ubiquitously expressed in a variety of tissues [202] and has been shown to be highly expressed in cardiac tissue [202,203] and to demonstrate cardioprotective and antiremodelling properties in a number of experimental settings. For example, with respect to cardioprotection, Metrnl has been implicated in cardioprotection due to enhanced angiogenesis in mice subjected to acute myocardial infarction [204]. These effects were shown to be mediated by activation of stem cell factor KIT proto-oncogene, receptor tyrosine kinase receptor (KIT), resulting in increased endothelial cell population in the infarct border zone [204]. While this study does not assign a direct antihypertrophic effect to Metrnl, it demonstrates a potential to reduce heart failure following infarction by enhancing cardiac repair. Moreover, Metrnl overexpression in cardiac macrophages resulted in substantial cardioprotection in mice subjected to myocardial ischemia and reperfusion, which reduced Metrnl expression on its own [205]. These protective effects were proposed to be mediated by increased macrophage polarization from M1 to M2 through the activation of AMPK phosphorylation, resulting in diminished release of proinflammatory factors [205]. Metrnl has also been shown to reduce cardiotoxicity in mice treated with the antitumour agent doxorubicin (Adriamycin). In this regard, cardiac-specific overexpression of Metrnl reduced oxidative stress, apoptosis, and cardiac dysfunction in these animals and improved survival while not affecting the antitumour properties of doxorubicin [206]. These beneficial effects were attributed to Metrnl-induced Sirtuin type 1 (SIRT1) activation via the cAMP/PKA pathway [206]. SIRT1 upregulation has also been shown to mediate the antihypertrophic effect seen in Metrnl-overexpressing mice subjected to thoracic aorta coarctation [207].

Metrnl is highly expressed in cardiac myocytes, and its expression has been shown to be dependent on PPARα activity [203]. Importantly, genetically generated Metrnl-deficient mice demonstrated enhanced adverse myocardial remodelling, as demonstrated by increased hypertrophy, fibrosis, and pro-inflammatory markers, as well as defective left ventricular function following 7-day isoproterenol infusion, effects which were reversed by Metrnl overexpression [203]. Although the precise mechanism underlying the antihypertrophic effect of Metrnl is unknown and requires further studies, these authors have demonstrated a potential role of p38-MAPK activation with subsequent phosphorylation of the transcription factor CREB, leading to the upregulation of the protective factor PGC-1α [203].

Another potential cellular process by which Metrnl could protect against myocardial remodelling is by targeting autophagy. For example, in both Type 1 and Type 2 diabetes mouse models, cardiomyopathy produced by hyperglycemic conditions was attenuated in animals with cardiac Metrnl overexpression, whereas Metrnl depletion exacerbated this condition [208]. These beneficial effects were associated with increased autophagy through stimulation of Unc-51-like autophagy-activating kinase 1 (ULK1), a key factor in autophagy initiation [209]. While this study implicates enhanced autophagy as a mechanism of protection against diabetic cardiomyopathy, protection against postinfarction myocardial remodelling by Metrnl was found to be associated with reduced autophagy. In this regard, cardiac Metrnl overexpression significantly reduced myocardial remodelling seen in hypertensive rats via autophagy inhibition, and Metrnl administration reduced angiotensin II-induced hypertrophy in cultured H9c2 myoblasts via BRCA2 DNA repair-associated (BRCA2)/Akt/mTOR signalling [210]. These effects were associated with reduced autophagy. Indeed, similar results were obtained in a mouse infarct model in which the subsequent myocardial remodelling was attenuated by myocardial Metrnl overexpression through a mechanism mediated by reduced autophagy [211]. Thus, inhibition of autophagy by Metrnl may represent a common mechanism for the antiremodelling effect of this adipokine.

The protective effect of Metrnl on myocardial remodelling and heart failure appears to be borne out in some clinical studies in that low plasma levels of this adipokine have been found to be associated with increased severity of heart failure in elderly patients [212]. However, post-infarction clinical studies suggest a potential deleterious role of this adipokine. Thus, an assessment of 400 patients with newly diagnosed heart failure revealed a higher incidence of all-cause and cardiovascular death associated with elevated serum Metrnl levels [213]. The reason for the apparent discrepancy is uncertain, but as suggested by others [203], the elevated plasma levels of this adipokine may reflect an attempted compensatory protective mechanism aimed at mitigating the heart failure process rather than a contributor to the pathology. Clearly, substantial further studies are necessary in order to clarify this issue and indeed the overall contribution of Metrnl to the heart failure process.

## 16. Progranulin

The 539 amino acid glycoprotein progranulin, first isolated from a tumorigenic cell line [214,215], is produced by various cell types but confirmed rather recently as an adipokine exerting a plethora of biological effects and participating in numerous diseases, particularly those involved in neural function [216]. It exerts anti-inflammatory properties, inhibits neutrophil activation, and reduces oxidative stress [217], and it has been shown to protect the ischemic and reperfused heart [218].

Although limited in scope, there is emerging evidence that progranulin inhibits myocardial remodelling and heart failure. From a general perspective, the ability of progranulin to limit the inflammatory response can bestow significant benefit in terms of attenuating cardiovascular pathology [219]. A specific contribution for this antiremodelling effect may be dependent on the ability of progranulin to reduce infarct size, as demonstrated when administered to mice subjected to sustained coronary artery occlusion [220], based on the fact that reduced infarct size would attenuate subsequent myocardial remodelling. Progranulin is abundantly expressed in mouse cardiac macrophages, whereas progranulin-deficient mice exhibited higher rates of mortality, increased left ventricular fibrosis, and increased arrhythmogenesis following 14-day coronary artery ligation, which was associated with alterations in macrophage-derived cytokine production, namely higher levels of TGF-β and interleukin (IL)-4R and lower amounts of IL-1β and IL-10 [221]. Moreover, in the absence of pathological insult, progranulin deficiency has been shown to enhance age-dependent cardiac hypertrophy as reported for 18-month-old mice via a mechanism involving complement C1q and activated β-catenin protein expression [222], key components of the wingless-related integration site (Wnt) signalling pathway, which can contribute to cardiac hypertrophy [223]. Indeed, blocking this pathway attenuated the cardiac senescence-associated increased hypertrophic response [222].

From patient studies, it is interesting to note that enhanced progranulin levels 7 days following acute myocardial infarction were associated with improved left ventricular function as assessed 6 months following infarction [224]. While suggestive of a salutary role of progranulin following myocardial infarction, this study has limitations in terms of a small patient population studied (18 subjects) and lack of evidence for a cause-and-effect relationship or mechanistic insights into the apparent benefit on left ventricular function.

## 17. Neuroregulin 4

Neuroregulin 4 (Nrg4) is an adipokine first identified in 1999 [225] and is a member of the epidermal growth factor family, which is widely expressed in brown adipose tissue as well as in many other tissues [226,227]. Nrg4 exerts its effects via the activation of the ErbB4 receptor tyrosine kinases and has been shown to participate in numerous physiological and pathophysiological processes [226,227].

There is recent emerging evidence that Nrg4 exerts cardioprotective effects, as has been shown in diabetes models of cardiomyopathy [228,229]. Moreover, antihypertrophic and antiremodelling effects of Nrg4 have been reported. For example, in a mouse model of 14-day isoproterenol infusion, myocardial remodelling was attenuated by a subsequent four-week Nrg4 treatment [230]. This effect of Nrg4 was associated with reduced hypertrophy, inflammatory response, fibrosis, and apoptosis and improved cardiac function [230]. These salutary effects were found to be dependent on the AMPK/NF-κB pathway [230]. Moreover, overexpression of Nrg4 attenuated myocardial fibrosis and cellular apoptosis in spontaneously hypertensive rats [231]. Using AC16 cardiomyocytes (a human ventricular myocyte cell line), Zhang et al. [232] recently reported that levels of Nrg4 can be increased following its stabilization by the RNA-binding protein Poly[RC] Binding Protein 2, thus resulting in reduced hypertrophy, oxidative stress, and inflammatory response following angiotensin II administration. Nrg4 exerts a myriad of beneficial effects, which could be conducive to indirectly limiting myocardial remodelling, including antioxidant and anti-inflammatory properties and reducing atherosclerosis [230,232,233]. Moreover, clinical observation showed that serum Nrg4 levels are inversely correlated with the severity of coronary artery disease [234]. The results thus far concerning Nrg4 have been promising in identifying a protective batokine limiting myocardial remodelling and heart failure, although substantial future work is required to fully delineate the role of Nrg4 in the heart failure process.

## 18. Retinol Protein Binding 4

Retinol protein binding 4 (RBP4) is an adipokine and transporter protein that exerts a variety of functions, including transport of vitamin A into the circulation. RBP4 generally is associated with deleterious effects, including increased insulin resistance, primarily by its ability to stimulate pro-inflammatory cytokines, thus contributing to the development of type 2 diabetes [235]. RBP4 is emerging as an important contributor to various pathologies related to cardiovascular disease, including promoting oxidative stress, apoptosis, and alterations in lipid metabolism and increasing insulin resistance, as already alluded to (reviewed in [236]).

Aside from its numerous effects promoting cardiac pathology, there is some evidence that RBP4 promotes cardiac hypertrophy through a number of cell signalling processes. For example, Gao and coworkers showed that RBP4 directly induces hypertrophy when added to mouse neonatal ventricular myocytes, an effect associated with increased expression of proinflammatory and oxidative stress markers [237]. The authors attributed the prohypertrophic effect of RBP4 to the activity of the TLR4/MyD88 pro-inflammatory pathway [237]. Although concrete clinical data are currently lacking, there is evidence that elevated levels of RBP4 are a predictor of adverse cardiac events in elderly patients with heart failure [238] or the development of cardiomyopathy in diabetic individuals [239].

## 19. CTRP Family

C1q/TNF-related proteins (CTRPs) represent a family of adiponectin paralogues exerting proinflammatory effects, which have been shown to modulate a myriad of conditions, including cardiovascular disease, such as coronary artery disease, via their ability to modulate the development of atherosclerosis (reviewed in [240,241]). However, the complexity of their actions is underscored by the fact that at least 15 of these proteins have been identified as possessing diverse biological effects, including diverse effects on myocardial remodelling and heart failure, acting through various cell signalling processes. A number of these proteins have been studied in terms of their ability to modulate the myocardial disease and the remodelling process in particular.

### 19.1. CTRP1

CTRP1 has not been extensively studied vis-à-vis myocardial remodelling, although current evidence suggests that it may contribute to myocardial remodelling by its ability to increase cardiac fibrosis. For example, CTRP1-deficient mice exhibited improved survival, reduced oxidative stress, and reduced inflammation following induction of myocardial infarction by suppressing macrophage activation through enhancing interaction between the adiponectin AdipoR1 and TLR4 [242]. In contrast, mice receiving CTRP1 injection exhibited worse outcomes following infarction [242]. On the other hand, CTRP1 deficiency has been shown to exacerbate angiotensin II-induced cardiac hypertrophy, fibrosis, inflammation, and oxidative stress associated with diminished cardiac function in mice through the activation of the AMPKα pathway [243].

As with many of the identified adipokines, contrary findings implicating CTRP1 as a pro-remodelling factor have also been reported through its ability to increase fibrosis. In this regard, mouse fibroblasts treated with recombinant CTRP1 demonstrated increased release of pro-inflammatory factors, whereas fibroblasts co-cultured with macrophages treated with recombinant CTRP1 demonstrated increased proliferation, effects attributed to activation of the NADPH oxidase 2/p38 pathway in macrophages [244].

### 19.2. CTRP3

CTRP3 has been shown to protect the ischemic and reperfused heart [245] and, more recently, to exert an antihypertrophic effect in mice subjected to thoracic aortic constriction. In this regard, CTRP3 knockout mice demonstrated enhanced hypertrophy and reduced left ventricular function 4 weeks post-aortic coarctation, whereas those animals overexpressing CTRP3 showed diminished cardiac hypertrophy and fibrosis and improved left ventricular function [245]. The beneficial effects of CTRP3 overexpression were attributed to inhibition of the p38/CREB pathway and a reduction in p38-induced endoplasmic reticulum stress [246]. Similarly to the antihypertrophic effect of CTRP3, we also observed in an identical model, which was accompanied by improved mitochondrial function and reduced oxidative stress [247]. These beneficial effects of CTRP3 overexpression were attributed to activation of the mitochondrial unfolded protein (UPRmt) response, a mechanism likely mediated by improved protein folding [247]. CTRP3 overexpression has also been shown to limit diabetic cardiomyopathy in rats produced by STZ administration or myopathy secondary to lipopolysaccharide-induced sepsis in mice through mechanisms involving activation of the AMPKα pathway [248,249]. Thus, overall evidence suggests that CTRP3 exerts an antihypertrophic and antiremodelling effect in various experimental models of heart failure. A contribution to the beneficial effect of CTRP3 may also reflect its ability to inhibit fibrosis. Indeed, this has been demonstrated in rats subjected to myocardial infarction and cultured cardiac fibroblasts, where CTRP3 overexpression attenuated myofibroblast differentiation and extracellular matrix production through AMPK-dependent mechanisms, resulting in inhibition of Smad3 activation myofibroblast differentiation [250].

It is noteworthy, however, that opposite effects of CTRP3 have also been reported. Thus, overexpression of CTRP3 resulted in enhanced myocardial hypertrophy and heart failure following 4-week thoracic aorta banding in mice by increased activation of the transforming growth factor-β-activated kinase 1 (TAK1)-JNK pathways, whereas CTRP3 knockdown exerted beneficial effects by inhibiting these signalling pathways [251].

### 19.3. CTRP6

Although there is a paucity of published data, there is evidence that CTRP6 exerts a beneficial effect in reducing heart failure. Heart failure produced by 2 weeks of isoproterenol injection in mice was attenuated by CTRP6 overexpression, as demonstrated by improved cardiac function and reduced apoptosis and mitochondrial reactive oxygen species through a mechanism mediated by the AMPK pathway [252]. As these effects were associated with diminished myocardial injury per se, it is possible that the beneficial effect was secondary to a cardioprotective influence of CTRP6 and not a direct antihypertrophic/antiremodelling effect of the protein. However, CTRP6 has been recently shown to have an antiremodelling effect in a rat infarction model primarily by its direct ability to attenuate fibroblast migration, thus contributing to an antifibrotic effect [253]. This influence of CTRP6 was attributed to AMPK and Akt activation targeting the RhoA/myocardin-related transcription factor-A (MRTF-A) pathway [253].

### 19.4. CTRP9

Few studies have been reported concerning CTRP9 with diverse implications on its role in myocardial remodelling or heart failure. In a rat model of right ventricular failure produced by sustained pulmonary artery banding, CTRP9 was shown to exert a protective effect by decreasing the production of reactive oxygen species and apoptosis in the right ventricle [254]. These effects were attributed to CTRP9-induced AMPK-dependent transcriptional activation of antioxidant enzymes, effects dependent also on adiponectin AdipoR1 and AdipoR2 receptor activation [254]. A beneficial effect of CTRP9 was further demonstrated in a model of cardiac hypertrophy in mice produced by administering a high-fat diet for 26 weeks. These animals developed cardiac hypertrophy and fibrosis, as well as endoplasmic reticulum stress-induced apoptosis, which were enhanced by CTRP9 knockout, as was left ventricular dysfunction, the latter being absent in wild type mice [255]. The authors proposed the LKB1/AMPK signalling pathway as the primary cellular mechanism underlying the beneficial effects of TRP9 [255].

In contrast to the beneficial effects reported, as noted above, Appari et al. have shown that CTRP9 overexpression exacerbates myocardial remodelling and enhanced cardiac dysfunction in a mouse model of pressure overload produced by thoracic aortic banding, whereas CTRP9 knockout mice were protected with a resulting diminished myocardial remodelling response [256]. From a mechanistic perspective, the authors implicated enhanced activated prohypertrophic ERK5 as the primary mechanism underlying the prohypertrophic effects of CTRP9 in response to pressure overload [256].

### 19.5. CTRP12

Although only one study has been reported, the findings suggest that CTRP12 protects against myocardial remodelling and the development of heart failure. Thus, CTRP12 overexpression reduced cardiac apoptosis, oxidative stress, and inflammation and improved left ventricular function in rats subjected for 6 weeks to myocardial infarction produced by coronary artery ligation, whereas CTRP12 deletion enhanced remodelling and worsened left ventricular dysfunction [257]. The salutary effects of CTRP12 overexpression were associated with activation of the TAK1-MAPK-JNK pathway in the protected hearts post-infarction [257].

### 19.6. CTRP15

Although CTRP15 has not been extensively studied, there is evidence, albeit limited, that it exerts antiremodelling effects. Thus, cardiac overexpression of CTRP15 in mice subjected to pressure overload-induced heart failure produced by sustained thoracic aortic banding resulted in reduced myocardial remodelling and improved cardiac function [258]. The beneficial effect of CTRP15 was attributed to its direct antifibrotic effect, as demonstrated in cultured cardiac fibroblasts [258]. Moreover, the antifibrotic effect of CTRP15 was shown to be mediated by insulin receptor (IR)/insulin receptor substrate-1 (IRS-1)/Akt pathway-dependent Smad3 activation [258].

**Figure 3:**
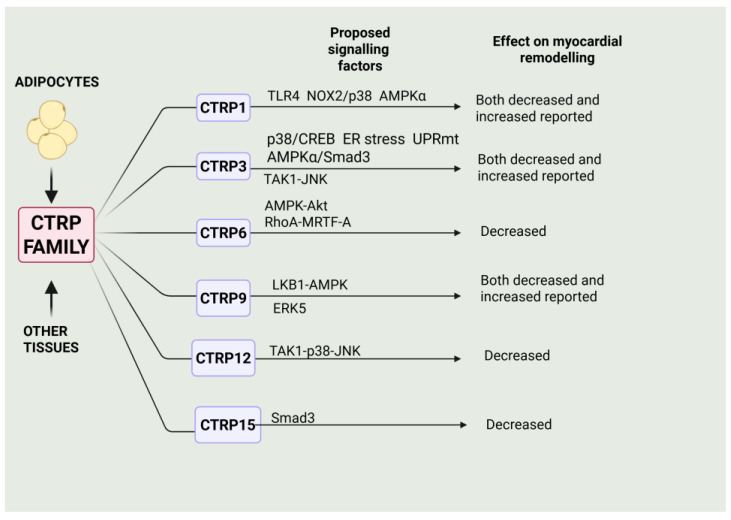
Summary of primary effects of some members of the CTRP family and the cell signalling factors implicated in these actions. Those factors exerting a protective effect are shown above the arrow, whereas the factors below the arrow depict factors mediating the adverse effects of myocardial remodelling for the respective CPRT protein. Please refer to the text for details. Created in BioRender. Karmazyn, M. (2025) https://BioRender.com/3jxxaoz.

Thus, in closing, it is clear that many members of the CTRP family exert profound effects on myocardial remodelling and heart failure, with a majority of these proteins demonstrating beneficial effects, albeit with some discrepant results. These are summarized in Figure 3.

## 20. Summary, Overall Conclusions, and Future Directions

**Figure 4:**
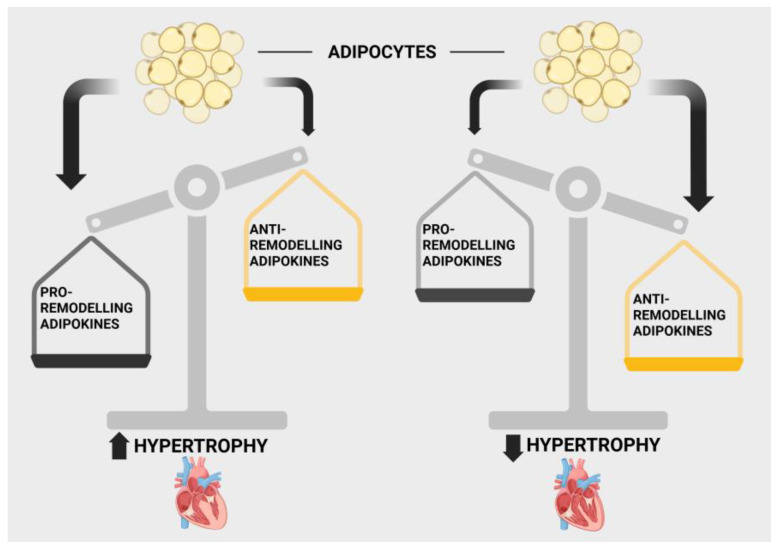
Illustration demonstrating the concept of balance between pro- and anti-remodelling adipokines in determining the resultant contribution to cardiac hypertrophy. Created in BioRender. Karmazyn, M. (2025) https://BioRender.com/ssxbe87.

The relationships between adipokine production and cardiac diseases, including those involving myocardial remodelling and heart failure, are complex and difficult to delineate. Among the reasons for such complexity is that many of these adipokines exert both beneficial and deleterious cardiac effects as discussed in this review, effects occurring via multifaceted molecular and cell signalling mechanisms. Moreover, as is evident from the present discussion, a multiplicity of cell signalling processes have been implicated for individual adipokines. While many adipokines exert pro-hypertrophic and pro-remodelling cardiac effects, at the same time, a large number of these proteins are considered as potential treatments for cardiovascular disorders [259]. In a recent extensive review, Packer outlined the role of adipokines in the context of their overall contribution to HFpEF, providing evidence for an adipose-driven disorder that dominates in importance when compared with the contribution of hypertension [260,261]. In his detailed and compelling discussion [260], Packer assigns adipokines to three specific categories: Domain I consisting of adipokines secreted primarily by healthy adipocytes that act as cardioprotective agents (e.g., adiponectin, omentin, Nrg4, CTRP3, CTRP9); Domain II adipokines secreted primarily by visceral adipocytes and which also exert cardioprotective and antiremodelling effects (e.g., vaspin, nesfatin, apelin, adipsin, progranulin); and lastly, Domain III adipokines synthesized primarily by hypertrophied and proliferating adipocytes seen in obese individuals, which contribute to cardiac inflammation and hypertrophy, resulting in HFpEF (e.g., chemerin, leptin, resistin) [260]. While this concept pertains primarily to HFpEF, the concept of selective and specific modulation of cardiac hypertrophy by adipokines can likely be applied to other cardiac pathologies. In view of the plethora of adipokines thus far identified, particularly those affecting the cardiac hypertrophic and remodelling processes, substantial further research is required to fully appreciate the contribution of specific adipokines to heart failure. What is clear is that a balance between beneficial and deleterious adipokines will determine their contribution to cardiac pathology including heart failure. This concept is summarized in Figure 4.

Challenging as this may be, understanding their contributions and the underlying cellular and molecular mechanisms can provide the opportunity for the use of selective adipokine blockers for the treatment of heart failure, particularly with heart failure associated with obesity. Moreover, the use of antiremodelling adipokines or their analogues offers the opportunity for additions to the currently available armamentarium for the treatment of heart failure.

## Figures and Tables

**Table 1:** Summary of studies demonstrating pro-hypertrophic/pro-remodelling effects of leptin in various experimental models and proposed underlying molecular and cellular mechanisms.

Experimental Model	Proposed Cellular Mechanisms	References
NRCM	p38 MAPK	[48]
NRCM	ET-1/ROS pathway	[49]
PHVM	JAK and MAPK pathways	[50]
NRCM	RhoA/ROCK	[51,52]
NRCM	mTOR-dependent RhoA/ROCK	[53]
NRCM	JAK/STAT activation	[54]
NRCM	RhoA/ROCK-caveolae upregulation	[55]
NRCM	PPARα activation	[56]
NRCM	calcineurin/NFAT	[57]
NRCM	JAK/STAT/CUX1-dependent FTO upregulation	[58]
Myocardial leptin overexpressing mice	TGF-β upregulation	[59]

NRCM, neonatal rat cardiac myocytes; p38 MAPK, p38 mitogen activated protein kinase; PHVM, pediatric human ventricular myocytes; ET-1, endothelin 1; ROS, reactive oxygen species; mTOR, mammalian target of rapamycin; RhoA/ROCK, Ras homolog gene family, member A/Rho-associated protein kinase; PPARα peroxisome proliferator-activated receptor α; NFAT, nuclear factor of activated T cells; CUX1, cut-like homeobox 1; FTO, fat mass and obesity-associated protein; TGF-β, transforming growth factor β.

**Table 2:** Summary of studies demonstrating anti-hypertrophic/anti-remodelling effects of adiponectin or adiponectin agonists in various experimental models and proposed primary underlying molecular and cellular mechanisms.

Experimental Model	Proposed Cellular Mechanisms	References
Mouse TAC	AMPK activation	[65]
NRCM	AMPK activation	[66]
STZ diabetic rat	Nrf2/Brg1 activation and HO-1 induction	[68]
Aged mouse DMD model	CAMKK2/pAMPK/PGC-1α activation	[69]
Mouse TAC	Cytokine reduction, other metabolic effects	[70]
STZ diabetic female rats	Increased Cx43 expression	[71]
Mouse TAC	AMPK activation	[72,73]
Ang II infusion [rat]	AMPK activation/MiR-133a upregulation	[74]
Aldosterone infusion in UNX mice	Reduced cytokines and oxidative stress	[75]
NRAM	AMPK activation	[76]
Ang II infusion (mouse)	Inhibition of β-catenin pathway	[77]

TAC, thoracic aorta constriction; STZ, streptozotocin; AMPK, AMP-activated protein kinase; Nrf2, nuclear factor erythroid 2-related factor 2; Brg1,Brahma related gene 1; DMD, Duchenne muscular dystrophy; HO-1, Heme oxygenase 1; CAMKK2, Calcium/calmodulin-dependent protein kinase 2; PGC-1α, peroxisome proliferator-activated receptor-gamma coactivator 1-alpha; Cx43, connexin 43; Ang II, angiotensin II; MiR-133a, microRNA 133a; UNX, uni-nephrectomized; NRAM, neonatal rat atrial myocytes.

## Data Availability

No new data were created or analyzed in this study. Data sharing is not applicable to this article.

## Abbreviations

The following abbreviations are used in this manuscript (in order of first mention):

WHO	World Health Organization
HFpEF	Heart failure with preserved ejection fraction
BMI	Body mass index
WATs	White adipose tissues
BATs	Brown adipose tissues
ObR (or LepR)	Leptin receptor
JAK2-STAT3	Janus Kinase 3-Signal Transducer and Activator of Transcription 3
NRCM	Neonatal rat cardiac myocytes
p38 MAPK	p38 mitogen activated protein kinase
PHVM	Pediatric human ventricular myocytes
ET-1	Endothelin 1
ROS	Reactive oxygen species
mTOR	Mammalian target of rapamycin
RhoA/ROCK	Ras homolog gene family, member A/Rho-associated protein kinase
PPARα	Peroxisome proliferator-activated receptor α
NFAT	Nuclear factor of activated T cells
CUX1	Cut-like homeobox 1
FTO	Fat mass and obesity-associated protein
TGF-β	Transforming growth factor β
AdipoR	Adiponectin receptor
ERK	Extracellular signal-regulated kinase
AMPK	AMP-activated protein kinase
TAC	Thoracic aorta constriction
STZ	Streptozotocin
Nrf2	Nuclear factor erythroid 2-related factor 2
Brg1	Brahma related gene 1
DMD	Duchenne muscular dystrophy
HO-1	Heme oxygenase 1
CAMKK2	Calcium/calmodulin-dependent protein kinase 2
PGC-1α	Peroxisome proliferator-activated receptor-gamma coactivator 1-alpha
Cx43	Connexin 43
Ang II	Angiotensin II
MiR-133a	MicroRNA 133a
UNX	Uni-nephrectomized
NRAM	Neonatal rat atrial myocytes
Mef2	Myocyte enhancer factor-2
TLR4	Toll-like receptor 4
MyD88	Myeloid differentiation factor 88
NF-κB	Nuclear factor kappa-light-chain-enhancer of activated B cells
miRNA	Micro-RNA
LKB1	Liver kinase B1 (also referred to as serine/threonine-protein kinase 11)
p70S6K	Ribosomal protein S6 kinase beta
NAMPT	Nicotinamide phosphoribosyltransferase
REV-ERB	Nuclear receptor subfamily 1 group D
PI3K	Phosphatidylinositol 3-kinase
JNK	c-Jun N-terminal kinase
TRPC1	Transient receptor potential canonical channel 1
APJ	Apelin receptor
AT1	Angiotensin II type 1 receptor
ACE	Angiotensin-converting enzyme
RAS	Renin angiotensin system
SGLT2	Sodium–glucose cotransporter-2
PEG	Polyethylene glycol
Akt	Protein kinase B
cAMP	Cyclic adenosine monophosphate
PKA	Protein kinase A
CCRL2	C-C chemokine receptor-like 2
Metrnl	Meteorin-like protein
KIT	KIT Proto-Oncogene, Receptor Tyrosine Kinase
SIRT1	Sirtuin type 1
ULK1	Unc-51-Like Autophagy-Activating Kinase 1
BRCA2	BRCA2 DNA repair-associated
IL	Interleukin
Wnt	Wingless-related integration site
Nrg4	Neuregulin 4
RBP4	Retinol protein binding 4
CTRP	C1q/TNF-related protein
UPRmt	Mitochondrial unfolded protein
TAK1	Transforming growth factor-β-activated kinase 1
MRTF-A	Myocardin-related transcription factor-A
IR	Insulin receptor
IRS-1	Insulin receptor substrate 1
